# Reversal of hypertensive heart disease: a multiple linear regression model

**DOI:** 10.15190/d.2021.17

**Published:** 2021-12-31

**Authors:** Shah Newaz Ahmed, Ratinder Jhaj, Balakrishnan Sadasivam, Rajnish Joshi

**Affiliations:** ^1^Department of Pharmacology, All India Institute of Medical Sciences, Bhopal, Madhya Pradesh, India; ^2^Department of General Medicine, All India Institute of Medical Sciences, Bhopal, Madhya Pradesh, India

**Keywords:** Hypertension, left ventricular hypertrophy, linear regression, renin angiotensin system, left ventricular mass.

## Abstract

Background: The development of left ventricular hypertrophy in primary hypertension increases cardiovascular mortality and morbidity. Reversal of left ventricular hypertrophy through therapeutic control of blood pressure reduces the risk of adverse cardiovascular incidents. Objective:  In our study, we explored for the determinants of left ventricular hypertrophy regression. Methods: A cohort (n=217) of patients with hypertensive left ventricular hypertrophy was identified by screening consecutive patients in medical outpatient unit. The primary inclusion criteria were (i) Blood pressure more than140/90 mm of Hg (ii) Left Ventricular Mass Index more than 115 and 95 gm/m2 for males and females respectively. Left Ventricular Mass Index was determined by echocardiography at the time of recruitment and after 24 weeks of standard pharmacotherapy. The change in Left Ventricular Mass Index was modelled using multiple linear regression with both categorical and continuous explanatory variables. The effect of drug therapy on change in Left Ventricular Mass Index was tested in the model with dummy coded variables for the treatment categories. Results: In simple linear regression, the variables significantly correlating with change in Left Ventricular Mass Index were baseline Left Ventricular Mass Index (r=0.62, p<0.001), change in systolic blood pressure (r=0.22, p=0.001), change in mean blood pressure (r=0.16, p=0.02), baseline systolic blood pressure (r=0.15, p=0.02), age (r=0.12, p=0.09) and diabetes (r=0.12, p=0.09). The best fit model (r2=0.408) retained baseline Left Ventricular Mass Index (β=0.59, p<0.001), change in systolic blood pressure (β=0.14, p=0.01) and diabetes (β=-0.104, p=0.05) as the significant predictors. Introduction of treatment effect into the model non-significantly increased the fit of the model (r2=0.414, p=0.27-0.98). Conclusions: Pre-treatment Left Ventricular Mass Index and reduction in systolic blood pressure were the major determinants of left ventricular hypertrophy regression. We also observed that there is lesser left ventricular hypertrophy regression in diabetic patients, warranting future research to explore glycaemic control as a modifiable factor in left ventricular hypertrophy reversal.

## INTRODUCTION

Essential hypertension is a major non-communicable chronic disease in our society and its incidence is expected to increase in near future. Progression of the disease leads to systemic involvement affecting vital organs including heart, brain and kidney. Association of the disease with increased incidence of cardiovascular events has been definitely proven^1^. Left ventricular hypertrophy (LVH), which develops as a consequence of increased blood pressure (BP), independently increases cardiovascular mortality and morbidity. It occurs in 36-41% of hypertensive patients^2,3^. Statistically, the mass of the left ventricle has a direct bearing on the incidence of cardiovascular accidents^1^. Adequate control of blood pressure retards the progression of the disease and regresses LVH, attenuating cardiovascular adversities^1,4-7^. Quantitatively, the decrease in blood pressure induces a proportionate decrease in Left Ventricular Mass (LVM)^8,9^. However, statistical models in contemporary literature could not completely explain the variability in LVM reduction. The gap in knowledge warrants further exploration of factors that may influence LVH regression. In our study, the predictors of LVM reduction were first identified individually by bivariate correlation. Using the significant correlates, we constructed a best fit multiple linear regression model by stepwise forward selection (SFS) method. We observed that 41 % of the variability in change in LVM was explained by the model. Our work can be a useful tool for health care professionals in assessment of response to therapy in primary hypertension.

## MATERIALS AND METHODS

### Study Site

The study was carried out at the medicine out-patient unit of a tertiary care centre in Central India.

### Ethical Approval

The study was enacted after obtaining ethical approval of the Institutional Human Ethics Committee, All India Institute of Medical Sciences, Bhopal.

### Study Subjects

Consecutive patients at the medicine outpatient unit were screened. Patients of either gender between 35 to 85 years old and blood pressure above 140/90 mm of Hg who had LVH were included in the study. LVH is defined as Left Ventricular Mass Index (LVMI) more than 115 and 95 g/m^2^ in males and females respectively. The exclusion criteria were 1. Renal pathology of any aetiology; 2. Pregnancy; 3. Pre-menopausal women willing to conceive; 4. Congestive heart failure NYHA class II- IV; 5. Morphological, congenital or electrical heart disease; 6. Left ventricular systolic dysfunction; 7. Bilateral renal artery stenosis; 8. Secondary hypertension; 9. Hepatic pathology of any aetiology. Only patients who provided voluntary informed consent were recruited in the research work. The data of 217 patients was available for analysis on completion of the study.

### Study Design

The data for the regression analysis was part of a larger non-randomized, open label, parallel group, prospective study designed to assess the quantum of LVM regression in hypertensive LVH following medical therapy^10^. All patients (N=217) received standard care for essential hypertension as per the existing guidelines. Both treatment-naïve and previously treated patients were recruited. Anti-hypertensive therapy comprised of Angiotensin converting enzyme inhibitors (ACEIs)/ Angiotensin receptor blockers (ARBs) monotherapy or combination of ACEIs/ARBs with the other two first line anti-hypertensive drugs viz. calcium channel blockers (CCBs) and thiazide diuretics (TD). On the basis of treatment regimen, four groups were constituted (Group A (n=70)- ACEIs/ARBs, Group B (n=48)- ACEIs/ARBs+CCB, Group C (n=51)- ACEIs/ARBs+TD, Group D (n=48- ACEIs/ARBs+CCB+TD). The dependent variable in the study was the change in LVMI following uninterrupted treatment for a period of six months.

### Clinical Tools

The standard protocol followed in this study for the echocardiographic estimation of LVM and measurement of blood pressure is described in our previously published work^10^.

### Model Development

The MLR model was constructed using SPSS (version 22). The response variable in the model was the quantum of change in LVMI following standard medical therapy. Both categorical and continuous predictors were tested for presumed addition as a significant variable in the model. Medical therapy comprising of four groups (Group A to Group D) was coded into four hierarchical treatment levels with Group A as the reference. At the end, the treatment levels were incorporated in the model as independent variables to test confounding effect due to medical therapy. The systematic method to develop the model is elaborated under the following steps.

#### Identifying the Individual Determinants of LVH Reversal:

The strength of correlation between change in LVMI (response variable) and the determinants of LVH reversal (the Predictors) was tested using bivariate linear regression. The categorical predictors were recoded into “0” and “1” and then tested for correlation. For instance, sex was represented as “1” and “0” for men and women respectively. In this preliminary step, the cut-off threshold probability was kept high (two tailed p value of < 0.1) to identify the maximum number of predictors for the dependent variable.

#### Adding Predictors into Model (Model Augmentation)

The threshold for feeding the correlates in the MLR model was also kept low (P≤ 0.1), to facilitate the inclusion of maximum number of variables in the model. The final inclusion of a significant variable in the MLR model was ascertained using SFS method. The method involves sequentially adding the predictors in decreasing order of the strength of correlation. After every addition, the fate of a predictor was decided using its significance level and increase in explained variability of the model.

#### Deleting Predictos from Model (Model Pruning)

A predictor was deleted from the model on basis of pre-set conditions after each iteration of SFS method. The cut off value of the level of significance for discarding a predictor from the model was set at > 0.1. A predictor was also deleted if there was multicollinearity between them. Multicollinearity was checked by correlation testing between a pair of variables. If the Pearson correlation coefficient was greater than 0.6, the stronger predictor was preferably retained in the model. We also checked for multicollinearity using Variance Inflation Factor (VIF). We set the cut off VIF at >3 to reject a variable

#### Construction of Best Fit Model

The final MLR model was created using those predictors only that remained after multiple iterations of the SFS method. The model was enacted with the preserved significant predictors. The coefficient of determination (R^2^) value was derived from the model. R^2^ denotes the fraction of the total variation in the outcome variable that can be explained by the regressors in the best fit model.

#### Independent MLR model for Treatment Effect (TE)

The best fit model did not account for the variability in change in LVMI due to drug treatment. We developed an MLR model independent of the best fit model to test the influence of treatment effect (TE) on change in LVMI. The four treatment categories (Group A to D) were converted into explanatory variables by coding into dummy variables using “0” and “1”. The reference Group A was coded as “0” only. The TE model was run with the four explanatory variables added en bloc by “Enter” method. R^2^ value was obtained for the model. The cut-off p value for statistical significance of the model as well as inter-group treatment effect difference was deemed as less than 0.05.

#### Forcing TE model into Best Fit Model

TE can be a confounding factor for the variables in the best fit model. We checked the confounding effect by combining the two models. The dummy variables for treatment categories were forced into the best fit model as explanatory variables. The change in R^2^ value and the statistical significance of the combined model were noted. The change in significance levels of intergroup difference in treatment effect was also noted.

#### Model Cross Validation

The MLR model was cross validated by both the Hold-out and Leave-one-out method^11^.

#### Hold-out Method

The study population (n=217) was divided into two successive sets-the training set (n_t_=145) and the validation set (n_v_=72). An MLR model predictive of change in LVMI was developed with the n_t_ and was used to predict the change in LVMI in the n_v_. Bivariate linear correlation between the actual and the modelled values of change in LVMI in the validation set was observed. 95% confidence interval of the slope of the regression line was calculated and the acceptable upper limit of the margin of error was kept at 10%. A P-value of < 0.05 was deemed statistically significant.

#### Leave-one-out Method

The predicted value for the change in LVMI was obtained for each subject using an iterative process. At each iteration, the predicted value of a particular subject was obtained using a best fit model derived using the data of all subjects except the one which was to be predicted. Bivariate linear correlation between the actual and modelled values of change in LVMI in the entire cohort was observed. 95% confidence interval of the slope of the regression line was calculated and the acceptable upper limit of the margin of error was kept at 10%. A P-Value of < 0.05 was deemed statistically significant.

## RESULTS

### Simple Linear Regression 

The variables selected and tested for correlation with change in LVMI for the sample population (n=217) are shown in Table 1. The significant variables were baseline LVMI (r=0.62, p<0.001), change in systolic BP (r=0.22, p=0.001), change in mean BP (r=0.16, p=0.02), baseline systolic BP (r=0.15, p=0.02), age (r=0.12, p=0.09) and diabetes (r=0.12, p=0.09).

### **M**ultiple Linear Regression 

**Figure 1:**
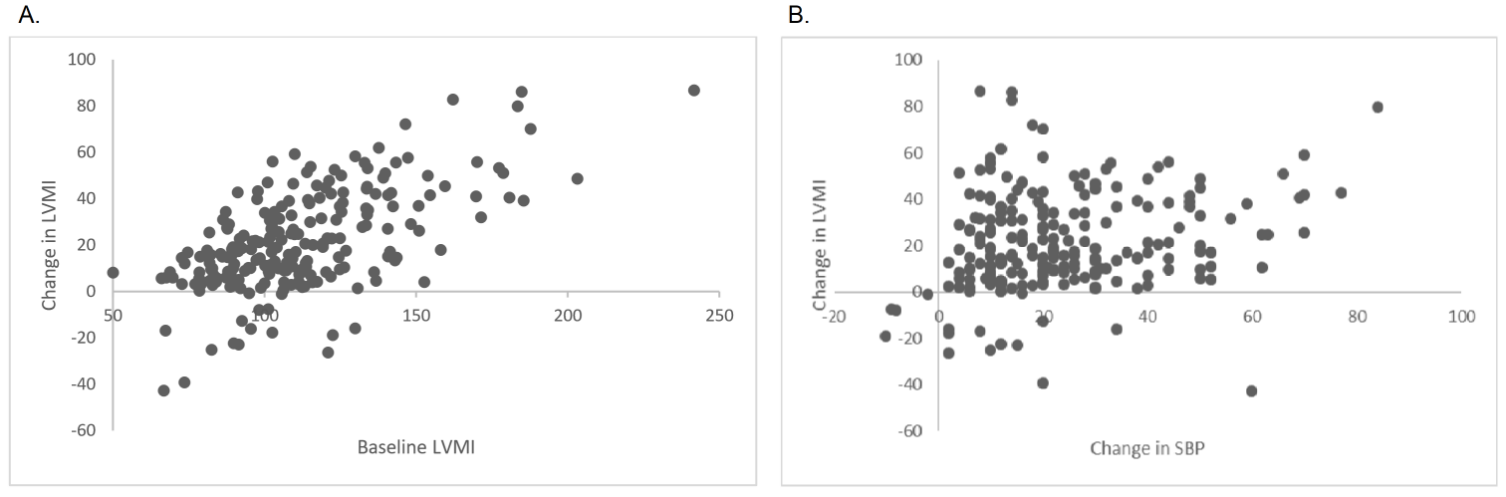
Diagram showing relationship of change in LVMI with the baseline LVMI (A.) and change in SBP (B.). LVMI - left ventricular mass index; SBP - systolic blood pressure

The proportion of explained variability (PEV) with baseline LVMI as the single predictor was 37.9% (r=0.616). PEV increased to 39.7% when change in systolic BP (SBP) was added as an explanatory variable in the model (P<0.05 for both the variables). When change in mean BP (MBP), baseline SBP (bSBP) and age were sequentially added to the model, PEV changed insignificantly (< 1.0%). These three variables which were statistically significant in simple linear regression became insignificant in the MLR model (P>0.05). However, baseline LVMI and change in SBP continued to be statistically significant at each step (P<0.05). There was an increase of 1.1% in PEV upon addition of diabetes as a predictor and the variable showed statistical significance (P=0.05). We also found that change in MBP and bSBP had very strong correlation with change in SBP (P=0.767 and 0.679 respectively) leading to redundancy. Therefore, in the MLR model, change in MBP and bSBP were dropped, while baseline LVMI, change in SBP and diabetes were retained. Further, we checked for interaction between the significant predictors by introducing their double products into the model. All such interaction were found to be statistically non-significant. In this way, the best fit model was finalized (R^2^=0.408) with baseline LVMI (β=0.59, p<0.001), change in systolic BP (β=0.14, p=0.01) and diabetes (β= -0.104, p=0.05) as predictors (Figure 1, A and B, Table 2).

**Table 1:** Predictors tested for addition to the multiple linear regression model for change in LVMI A two tailed P value<0.1 was deemed statistically significant. LVMI- Left ventricular mass index, CI- Confidence interval, SD-Standard deviation, BP- Blood pressure

Predictor	Mean±SD/ Ratio	Correlation coefficient	Unstandardized coefficient (95% CI)	P value
Age (years)	56.53±11.58	0.12	0.21 (-0.04-0.46)	0.09
Gender (male/female)	134/83	0.04	-1.69 (-7.6-4.2)	0.58
Duration of hypertension (years)	5.20±5.65	0.08	0.28 (0.22-0.78)	0.27
Treatment naive (yes/no)	39/178	0.06	3.52 (-3.97-11.01)	0.36
Ischemic heart disease (yes/no)	56/161	0.01	0.66 (-6.06-7.37)	0.85
Diabetes (yes/no)	78/139	0.12	-5.17 (-11.14-0.80)	0.09
Dyslipidaemia (yes/no)	28/189	0.04	-2.34 (-10.96-6.23)	0.59
Baseline systolic BP (mm of Hg)	161.01±18.77	0.15	0.18 (0.02-0.33)	0.02
Baseline diastolic BP (mm of Hg)	93.17±11.77	0.001	-0.002 (-0.25-0.24)	0.99
Baseline mean BP (mm of Hg)	115.78±11.67	0.08	0.15 (-0.10-0.40)	0.23
Change in systolic BP (mm of Hg)	23.50±17.32	0.22	0.27 (0.11-0.44)	0.001
Change in diastolic BP (mm of Hg)	9.20±11.15	0.06	0.12 (-0.14-0.37)	0.38
Change in mean BP (mm of Hg)	13.94±10.86	0.16	0.31 (0.05-0.58)	0.02
Baseline pulse rate (beats/min)	81.31±13.09	0.01	-0.02 (-0.24-0.20)	0.84
Change in pulse rate (beats/ min)	2.12±8.95	0.005	0.01 (-0.31-0.34)	0.94
Baseline LVMI (g/m2)	111.99±28.26	0.62	0.47 (0.39-0.55)	<0.001
Relative wall thickness	0.65±0.13	0.05	-8.25 (-30.0-12.50)	0.43

### Influence of Treatment Effect (TE) 

**Figure 2:**
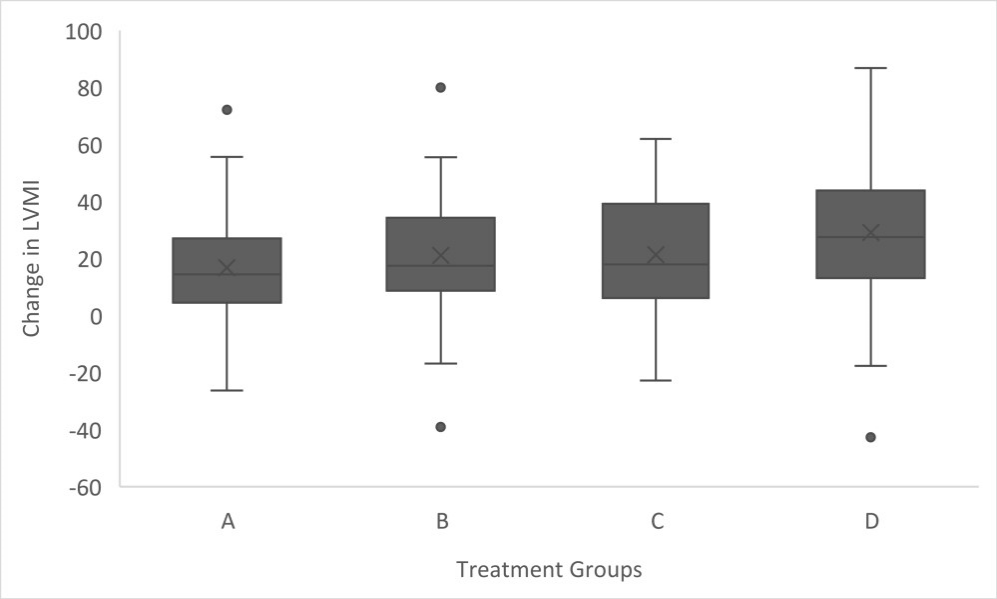
Box and Whisker plot representing mean change in LVMI with 95% confidence interval in the four treatment groups LVMI- Left ventricular mass indexLVMI - left ventricular mass index; SBP - systolic blood pressure.

The independent MLR model for treatment effect was statistically significant (R^2^=0.044, P=0.022) with significant TE difference between Group A and Group D (P=0.002) (Figure 2). Upon forcing the TE model with the best fit model into a combined model, PEV increased by 0.6% only (from 40.8 to 41.4 %) and the significant difference between Group A and D became non-significant (P value changed from 0.002 to 0.27). This indicates that the predictors in the best predictive model are the primary determinants of change in LVMI irrespective of the category of anti-hypertensive drug therapy.

### Cross Validation

#### Hold-out Method

**Figure 3:**
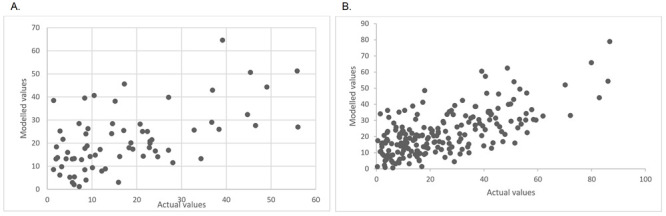
Plot of bivariate correlation between the actual and the modelled values of change in LVMI in the hold-out validation method (A.; R=0.59) and the leave-one-out validation method (B.; R=0.64) respectively. The negative values of the variables are not shown in graph.

The mean change in LVMI in the n_t_ was 23.92 ±23.55 gm/m^2^. The mean values of the actual and the modelled change in LVMI in the n_v_ was 16.54±15.63 and 20.46±13.40 gm/m^2^ respectively. The Pearson coefficient for bivariate correlation between the actual and the modelled values of change LVMI in the n_v_ was 0.59 (P<0.001) (Figure 3). The margin of error of the slope of the regression line (0.393, 95% confidence interval: 0.283-0.504, P<0.001) was 10.9%.

#### Leave-one-out Method

The mean of change in LVMI in the whole set and leave-one-out set was 21.47 ±17.67 and 21.47±13.73 gm/m^2^ respectively. The Pearson coefficient for bivariate correlation between the actual and the modelled values of change LVMI in the two sets was 0.64 (P<0.001) (Figure 3). The margin of error of the slope of the regression line (0.408, 95% confidence interval: 0.342-0.474, P<0.001) was 6.6%.

**Table 2:** Stepwise forward selection method for development of predictive model for change in LVMI LVMI-Left Ventricular Mass Index, SBP-Systolic Blood Pressure, MBP-Mean Blood Pressure, VIF-Variation inflation factor, R2-Coefficient of determination.

Step	Predictors added	P value	VIF	R2	Adjusted R2	Predictors removed
1	Baseline LVMI	<0.001	1.00	0.379	0.376	
2	Baseline LVMI	<0.001	1.02	0.397	0.392	
	Change in SBP	0.012	1.02			
3	Baseline LVMI	<0.001	1.02	0.399	0.390	Change in MBP
	Change in SBP	0.032	2.44			
	Change in MBP	0.49	2.43			
4	Baseline LVMI	<0.001	1.05	0.407	0.399	Baseline SBP
	Change in SBP	0.002	1.85			
	Baseline SBP	0.062	1.91			
5	Baseline LVMI	<0.001	1.04	0.398	0.389	Age
	Change in SBP	0.014	1.03			
	Age	0.674	1.02			
6	Baseline LVMI	<0.001	1.02	0.408	0.400	
	Change in SBP	0.01	1.02			
	Diabetes	0.05	1.00			

## DISCUSSION

Regression of LVH is the surrogate marker for the efficacy of antihypertensive therapy in reducing cardiovascular events. Therefore, the factors determining LVH regression are of immediate clinical interest. In our work, we explored the tentative variables that could have a possible relation with the quantum of LVH regression. We incorporated the significant variables into a single multiple linear regression model. The objective of multiple linear regression modelling is to integrate the significant regressors in a single viable model to achieve the best possible prediction for the outcome of interest. It seeks to get rid of the spareable predictors and optimizes the effect of the individual regressors into a common final value. Validated models can help in rapid and correct assessment of clinical and diagnostic outcomes. We developed the MLR model predictive for LV mass regression using stepwise forward selection method.

Our Best Fit Model included the most important variables and excluded the redundant variables. The three variables that found place in the Best Fit Model were baseline LVMI (β=0.59, p<0.001), change in systolic BP (β=0.14, p=0.01) and diabetes (β= -0.104, p=0.05). The model explained 40.8% of the variability in change in LVMI. We compared our results with a similar study conducted by Konstantinos et al. The researchers had developed an MLR model by “stepwise backward method” which could explain 36% of the variability in change in LVMI. Baseline LVMI and change in systolic BP were the common predictors. In addition, the study reported “impaired left ventricular geometry and blunted nocturnal blood pressure fall before treatment” as significant predictors of LVH regression. The researchers did not find any correlation with diabetes^12^. However, it is known that hypertensives with diabetes have greater LV mass^13-15^. Diabetics also show lesser regression of LVH with the same decrease of blood pressure compared to non-diabetics. In the Losartan Intervention For Endpoint (LIFE) Reduction in Hypertension Study, the regression in LVH was studied in 9193 hypertensives (of which 1195 were diabetics) using electrocardiography (ECG) Cornell voltage-duration product criteria. At the end of 5 years, the decrease in LVH by the ECG criteria was less in diabetics (P<0.001)^16^.

In our study, we used echocardiography to measure LV mass and got similar finding that LVH regression was less in diabetics (unstandardized coefficient= -4.64). The Aliskiren in Left Ventricular Hypertrophy (ALLAY) trial which employed magnetic resonance for cardiac mass also reported that diabetes mellitus (P = 0.043), change in systolic blood pressure (P = 0.005) and pre-treatment LVMI (P < 0.001) are significant predictors of LVH regression^17^.

However, further studies are warranted to explore the effect of glycemic variability in regression of hypertensive LVH. In a cohort of patients having concurrent diabetes and hypertension, it was found that more intensive lowering of BP causes greater regression of LVH^17^. In the ACCORD BP trial, intensive and standard BP lowering (target SBP of <120 and <140 mmHg respectively) were compared in 4733 diabetic patients (SBP 130-80 mm of Hg) not receiving more than 3 drugs for hypertension. There was greater regression of already existing LVH and also lesser development of new LVH. It was assumed that lower risk of LVH will have the potential to reduce cardiovascular mortality and morbidity^18^. The SPRINT trial was a randomized multicenter trial conducted on 8164 hypertension patients without diabetes which also compared the outcomes of intensive BP lowering with standard BP lowering. It not only evaluated the risk of LVH but also compared the rates of CVD events in the two arms. Similar to the ACCORD BP trial, it was found that there was greater chance of regression of previous LVH and lesser chance of development of new LVH with intensive BP lowering. The risk of cardiovascular accidents was found to be 24% lower (P value=0.001) in the intensive BP lowering group. However, the researchers found that the reduction in CVD events was over and above of what could be assigned to the favorable effect of LVH regression. It was concluded that intensive BP reduction had cardiovascular benefit that go beyond the effect of hemodynamic stress on the heart^19^. A metanalysis of randomized controlled trials performed by Schmieder et al in 1996 observed that the decrease in LVMI was related with the decrease in blood pressure (systolic R=0.46, P<0.001; diastolic R=0.21, P=0.08)^8^.The outcome of another metanalysis by Jennings et al similarly concluded that there is a “strong relationship between changes in blood pressure and LVH regression”^9^.

Our MLR model also indicates that the single most important factor amenable to intervention is the reduction in systolic blood pressure. Adequate control of blood pressure will not only retard the progression of the disease but also regress left ventricular hypertrophy, reducing the rate of cardiovascular complications. However, the drug regimens practiced achieving these therapeutic objectives are empirical based on limited studies. Amongst the different antihypertensives, ACE inhibitors and ARBs are considered first line drugs for therapy in hypertensive heart diseases. Inhibition of the Renin-angiotensin aldosterone system by ACE inhibitors/ ARBs is the most accepted theory that explains their beneficial effect^20,21^. We studied the role of clinical factors in inducing left ventricular regression primary hypertension patients receiving ACE inhibitor/ ARB based regimen. The treatment arms in our observational study were non-randomized, naturally occurring groups based on convenience sampling and the treatment effects are not transferable across study cohorts. An MLR model is a viable alternative method to find out the predictors of the outcome of interest in such a non-experimental study. In our model, we did not find any novel predictor for LVH regression. However, we have elaborated a systematic approach towards construction of the predictive model. We tested and statistically controlled confounding due to treatment effect. This enabled us to identify the deterministic factors independent of the treatment categories. We also performed an inter-group comparison (one way ANOVA) between the four groups with respect to change in BP and LVMI (results shown in our previous work ^10^). We found that in mutually exclusive groups and not meriting statistical comparison, combination therapy brings greater reduction in BP as well as greater regression of LV mass. It is such that the maximum regression is seen in the group on combination of three drugs. When change in BP was introduced as a covariate (ANCOVA method), there was no statistically significant difference between the groups^10^.

The results of ANCOVA and MLR model are in congruence with each other. Both analyses indicate that BP control is the modifiable factor amenable to treatment in regression of hypertensive LVH. The proportion of change in LV mass that can be attributed to either drug or BP reduction are inter-related and inseparable because BP reduction itself varies as a function of the drug class and dose. Therefore, the objective of antihypertensive therapy would be to control blood pressure through rational use of the recommended drugs. This observation asserts the recommendations of recent guidelines of prompt shifting to combination therapy of antihypertensives for better control of blood pressure and long-term prognosis. The results also reinforce the recommendation of existing guidelines that triple drug therapy should be used to reach the goal of target blood pressure whenever indicated^21-23^.

## CONCLUSION

While pre-treatment left ventricular mass is the major determinant of left ventricular hypertrophy regression, the only factor amenable to standard treatment is reduction in systolic blood pressure. Our study also shows that there is lesser left ventricular hypertrophy regression in diabetics warranting future research to explore glycaemic control as a modifiable factor in left ventricular hypertrophy reversal.
